# Valorization of Brewing By-Products for Sustainable Active Material

**DOI:** 10.3390/foods15122141

**Published:** 2026-06-13

**Authors:** Luciana B. Malbos, Paula Garcia-Oliveira, Irene T. Seoane, Jesus Simal-Gandara, Liliana B. Manfredi, Viviana P. Cyras, Lucía Cassani

**Affiliations:** 1 Instituto de Investigaciones en Ciencia y Tecnología de Materiales (INTEMA), Facultad de Ingeniería, Universidad Nacional de Mar del Plata—Consejo de Investigaciones Científicas y Técnicas (CONICET), Mar del Plata 7600, Argentina; lucianamalbos@fi.mdp.edu.ar (L.B.M.); itseoane@fi.mdp.edu.ar (I.T.S.); lbmanfre@fi.mdp.edu.ar (L.B.M.); vpcyras@fi.mdp.edu.ar (V.P.C.); 2 Departamento de Ingeniería Química y en Alimentos, Facultad de Ingeniería, Universidad Nacional de Mar del Plata, Mar del Plata 7600, Argentina; 3 Universidade de Vigo, Departamento de Química Analítica y Alimentaria, Facultad de Ciencias, 32004 Ourense, Spain; paula.garcia.oliveira@uvigo.gal (P.G.-O.); jsimal@uvigo.es (J.S.-G.); 4 Instituto de Agroecoloxía e Alimentación (IAA), Universidade de Vigo, Campus Auga, 32004 Ourense, Spain; 5 Agricultural and Food Research Group (AA1), Galicia Sur Health Research Institute (IIS Galicia Sur), SERGAS-UVIGO, 36312 Vigo, Spain

**Keywords:** brewer’s spent grain, pressurized liquid extraction, response surface methodology, starch, active packaging

## Abstract

Brewer’s spent grain (BSG), the main by-product of the brewing industry, is an abundant lignocellulosic residue that remains underused. In this study, antioxidant-rich extracts were obtained from BSG using pressurized liquid extraction (PLE) and subsequently incorporated into thermoplastic starch (TPS) films for sustainable food packaging applications. The phenolic profile analysis revealed 13 compounds, with caffeic acid and its hexoside as the most abundant. Extraction conditions were optimized using response surface methodology (RSM) to maximize yield and total phenolic content, showing that temperature had a significant positive effect. The selected extract had a total phenolic content of 3.19 mg/g dw and exhibited notable antioxidant activity. It was then incorporated into the polymer matrix, and the resulting films were analyzed for their structural, thermal, and antioxidant properties. The incorporation of BSG extracts improved the film antioxidant activity. Additionally, the release of phenolic compounds was evaluated and successfully described using a diffusion model based on Fick’s law, which allowed the calculation of a diffusion coefficient D = 2.63 × 10^−8^ cm^2^/s. Overall, the findings indicate that BSG-based extracts may represent promising functional additives for biodegradable polymer films, and the developed TPS films serve as proof-of-concept active packaging materials from renewable agro-industrial residues.

## 1. Introduction

Every year, around 30 million metric tons of brewer’s spent grain (BSG) are generated worldwide, making it one of the most abundant industrial by-products [1]. In Argentina, beer production reached about 20 million hectoliters in 2022, which translates into about 400,000 tons of BSG, assuming an average of 20 kg per hectoliter [2]. Despite these large amounts, BSG is still mostly discarded or used in low-value applications such as animal feed or fertilizer.

BSG is the main by-product of the brewing process and represents about 85% of the total waste generated [3]. It is produced during the mashing step, when malted cereals, mainly barley, are mixed with water to activate enzymes that break down starch and proteins. This process produces wort, the liquid used for fermentation, and a solid residue known as BSG [4].

Because of its availability and composition, BSG is a promising raw material for valorization and could help improve the sustainability of the brewing industry. It has already been studied for uses such as energy production, incorporation into food as a source of dietary fiber, and recovery of phenolic compounds with functional properties [5]. However, there are still few studies focused on extracting phenolic compounds from BSG specifically for use in sustainable packaging materials. This limits the development of more circular approaches that connect waste valorization with biodegradable packaging.

Phenolic compounds are of particular interest because of their antioxidant activity and their ability to slow down oxidative degradation in foods [6]. Compounds such as flavonoids, phenolic acids, and tannins can neutralize free radicals, helping to reduce lipid oxidation and preserve food quality [7]. Since oxidation often occurs at the interface between food and its surroundings, incorporating natural antioxidants into packaging materials has become an attractive strategy to extend shelf life.

To recover these compounds, this study uses pressurized liquid extraction (PLE), a technique that is considered more environmentally friendly than conventional methods. It allows shorter extraction times, uses less solvent, and generally improves efficiency. Another advantage is that it works well with green solvents like water and ethanol, which were used here. The efficiency of PLE depends on factors such as temperature, extraction time, and solvent composition, so Response Surface Methodology (RSM) was applied to optimize these conditions and maximize both phenolic recovery and extraction yield. While phenolic extraction from BSG has been studied using other techniques [8,9,10], its application using PLE has not been widely explored.

At the same time, the food packaging sector accounts for nearly 40% of global plastic consumption, contributing significantly to environmental pollution [11]. As a result, biodegradable materials derived from renewable resources have gained attention as sustainable alternatives. Thermoplastic starch (TPS), produced by plasticizing native starch under heat and shear in the presence of plasticizers such as water or glycerol, stands out due to its low cost, biodegradability, and wide availability [12,13,14]. Additionally, advantages such as thermal stability and char-forming capacity further enhance its suitability for various applications [15].

TPS films can also be functionalized with bioactive compounds to enhance their properties. Previous studies have incorporated additives such as green tea extract, olive oil, carvacrol nanoemulsions, and agricultural residues, improving barrier performance, antioxidant activity, and antimicrobial properties [16,17,18,19].

In this context, the present study combines both approaches by incorporating antioxidant-rich extracts obtained from BSG via PLE into TPS films produced by casting. The release of phenolic compounds into an alcoholic food simulant was evaluated to better understand their release behavior and potential application in active food packaging.

## 2. Materials and Methods

### 2.1. Materials

BSG was kindly provided by the brewery Cerveza Baum (Mar del Plata, Argentina, https://cervezabaum.com/#fabrica, accessed on 1 June 2026) from a single production batch of a pale beer prepared with Pilsner-type barley malt. Potato starch was supplied by Avebe Argentina S.A. (Buenos Aires, Argentina), and glycerol (≥99.5% purity, molar mass 92.09 g/mol) was purchased from Anedra (Buenos Aires, Argentina).

For the different assays, the following reagents were used: 2,2-diphenyl-1-picrylhydrazyl (DPPH) (Alfa Aesar, Thermo Fisher, Kandel, Germany), 2,2′-azino-bis(3-ethylbenzothiazoline-6-sulfonic acid) (ABTS) (Alfa Aesar, Thermo Fisher, Kandel, Germany), potassium persulfate (Carlo Erba Reagents, Milan, Italy), Folin–Ciocalteu reagent (VWR Chemicals, Darmstadt, Germany), and sodium carbonate (Na_2_CO_3_) (Carlo Erba Reagents, Milan, Italy). Ethanol (96%) and acetonitrile (HPLC grade) were used as solvents. Formic acid (Carlo Erba Reagents, Milan, Italy) was used to acidify the mobile phases. The following analytical standards used for mass spectrometry identification were purchased from Sigma-Aldrich (St. Louis, MO, USA): gallic acid, caffeic acid, ferulic acid, apigenin, catechin, and quercetin.

### 2.2. Pressurized Liquid Extraction (PLE)

BSG was freeze-dried to a final moisture content of 7% (*w*/*w*) and subsequently milled and sieved through a 600 µm mesh to obtain a homogeneous particle size. The dried and sieved material was thoroughly mixed before sampling. PLE was carried out using an accelerated solvent extractor (Dionex™ ASE™ 350, Sunnyvale, CA, USA). For each run, 1 g of BSG was loaded into a 22 mL stainless-steel cell between two layers of Ottawa sand, with a cellulose filter at the bottom. Water–ethanol mixtures were used as solvents, and extracts were collected in 250 mL bottles. A total of 28 extractions were performed following a design matrix combining extraction time (5–30 min), temperature (50–200 °C), and solvent composition (0–100% ethanol). Each run included a standard heat-up step prior to extraction. After extraction, samples were centrifuged at 6000 rpm for 15 min, filtered through a 0.22 μm nylon syringe filter, and stored at −20 °C in the dark until LC–MS/MS analysis. The solid residue remaining after extraction was not discarded; instead, it was further processed to produce a cellulose-based cardboard-like material, providing an additional valorization route for BSG.

### 2.3. Phenolic Compounds Profile of BSG Extracts

The identification of phenolic compounds was performed using LC-MS/MS. For this purpose, the phenolic profile was analyzed using a Dionex Ultimate 3000 UPLC+ system (Thermo Scientific, Waltham, MA, USA) coupled to a TSQ Quantis triple quadrupole mass spectrometer (Thermo Scientific, USA). Chromatographic separation was carried out on a Nucleosil C18 column (3 μm, 100 Å, 150 × 4.6 mm, Phenomenex, Torrance, CA, USA) kept at 35 °C. The mobile phases consisted of Milli-Q water with 0.1% formic acid (A) and acetonitrile (B), following the gradient: 15% B (5 min), 15–30% B (10 min), 30–55% B (10 min), 55–80% B (10 min), 80% B (10 min), and finally 15% B (10 min) for re-equilibration, for a total run time of 55 min. The flow rate was 0.5 mL/min, and the injection volume was 50 µL. Mass spectrometric detection was performed using an electrospray ionization (ESI) source in negative mode and acquired in selected reaction monitoring (SRM) mode, where precursor-product ion transitions enable targeted detection of phenolic compounds. Universal source parameters were sheath gas 30 Arb, auxiliary gas 10 Arb, ion transfer tube temperature 325 °C, and vaporizer temperature 350 °C. Data acquisition and interpretation were conducted using FreeStyle software (1.8 SP1, ThermoFinnigan, San Jose, CA, USA).

When available, phenolic compounds were identified by comparing their retention times and mass spectra with those of pure analytical standards: gallic acid, caffeic acid, ferulic acid, apigenin, catechin, and quercetin. When commercial standards were not available, tentative identification was performed by comparing the acquired information with previously reported data [20,21,22]. After characterization, the compounds present in the BSG extract were systematically categorized into phenolic classes and subclasses (Table 1).

Quantification of phenolic compounds in the BSG extracts was performed using calibration curves constructed for each available analytical standard. For compounds identified without reference standards, quantification was estimated using the calibration curve of the most structurally similar standard. Total phenolic content (TPC) was calculated as the sum of the quantified compounds and expressed as milligrams per gram of dry weight (mg/g dw).

### 2.4. Response Surface Methodology for Extraction Optimization

#### 2.4.1. Experimental Design and Mathematical Modelling

The experimental design consisted of a three-factor, five-level central composite design combined with a factorial design, resulting in a total of 28 experiments. The complete design matrix, including both the coded and actual values of the independent variables, is presented in Table 2.

Two response variables were selected for this study:

- Extraction yield (Y_1_), determined gravimetrically according to the method described by Cassani et al. [23]. Briefly, 5 mL of extract were placed in pre-weighed crucibles and dried in an oven at 104 °C until complete solvent evaporation. The dry extract mass was calculated as the difference in weight before and after drying. The procedure was performed in duplicate, and the results were expressed as g extract/100 g dw (% *w*/*w*).
- Total phenolic content (TPC or Y_2_), calculated as the cumulative sum of individual phenolic compounds quantified by chromatography, as described in Section 2.3 and expressed as mg TP/g dw.

The experimental data for each response were fitted to second-order polynomial models (Equation (1)) using the least squares method:

(1) $$Y_{n}=\beta_{0}+\sum_{i=1}^{n}\beta_{i}X_{i}+\sum_{i=1}^{n-1}\sum_{j=2}^{n}\beta_{\mathrm{ij}}X_{i}X_{j}$$

where Y_n_ is the predicted response variable, X_i_ is the dimensionless coded value of the independent variable, β_0_ is the constant coefficient, β_i_ is the linear coefficient, β_ij_ is the interaction effect coefficient, and n is the number of variables considered in the analysis.

#### 2.4.2. Simultaneous Optimization and Validation

The desirability function was applied to perform simultaneous multi-response optimization [24]. In this approach, TPC was set with the highest relative importance (value of 5), while extraction yield was also maximized but assigned a lower relative importance (value of 3), considering that yield reflects the total mass of dry extract recovered, which may include non-phenolic constituents.

To validate the optimization results, an additional set of extractions was carried out using the optimal operating conditions predicted by the desirability analysis.

### 2.5. Antioxidant Activity of BSG Extract Obtained in Optimal PLE Conditions

The antioxidant activity of the extract obtained under optimized conditions was evaluated using two assays: the radical scavenging capacity of DPPH and the radical scavenging capacity of ABTS.

#### 2.5.1. DPPH Assay

For the DPPH assay, extracts were diluted in ethanol to obtain seven serial dilutions. Aliquots of 50 µL were mixed with 200 µL of DPPH solution (225 µM) in a 96-well microplate. A control was prepared using ethanol instead of sample. Absorbance at 517 nm was measured immediately (t = 0) and after 60 min of incubation at room temperature in the dark using a Synergy HTX multi-mode reader (Bio-Tek, Winooski, VT, USA). Trolox was used as a positive control. The radical scavenging capacity was expressed as the percentage of inhibition (%I), calculated using Equation (2),

(2) $$\%I=\frac{A_{c,0}-A_{s,60}}{A_{c,0}}\;\times \;100\%$$

where A_c,0_ is the initial absorbance of the control and A_s,60_ is the absorbance of the sample at 60 min.

DPPH scavenging activity (%) was plotted against extract concentration (mg/mL) and the data were fitted to a Weibull model (Equation (3)).

(3) $$Y=k\times (1-e^{-\ln2\times {\left(\frac{X}{{\mathrm{IC}}_{50}}\right)}^{a}})$$

where Y is the observed inhibition (%I), X is the extract concentration, k is the maximum response (upper asymptote), IC_50_ is the concentration required to inhibit 50% of the DPPH radical, and a is a curve-shape parameter describing asymmetry and curvature.

#### 2.5.2. ABTS Assay

For the ABTS assay, the ABTS^•+^ radical was generated as described by Cassani et al. [23]. Aliquots of 50 µL of each extract dilution were mixed with 200 µL of ABTS^•+^ solution in a 96-well microplate. A control was prepared using ethanol instead of sample. Absorbance at 734 nm was recorded immediately (t = 0) and after 60 min of incubation at room temperature in the dark. Trolox was used as a positive control. Radical scavenging activity (%I) was calculated using Equation (2). Inhibition values were plotted against extract concentration, and IC_50_ values were obtained by fitting the data to the Weibull model (Equation (3)).

### 2.6. Preparation of TPS Film Containing BSG Extract

Active TPS films were prepared by casting. Briefly, 2 g of lyophilized extract were dissolved in 15 mL of distilled water. Separately, 2 g of starch and 1 g of glycerol were dispersed in 30 mL of water and stirred for 15 min at room temperature. The selected extract loading was used as a proof-of-concept formulation to ensure detectable phenolic release and to evaluate migration behavior from the starch-based matrix. The mixture was then heated to 90 °C and maintained under stirring for 15 min to ensure complete gelatinization. Afterward, 15 mL of the extract solution were added, and the mixture was stirred for 20 min in an orbital shaker to remove air bubbles. The resulting gel was cast into plastic Petri dishes and dried at 50 °C for 24 h to obtain uniform films.

#### 2.6.1. Fourier Transform Infrared Spectroscopy (FTIR)

TPS films with and without extract, as well as the extract alone, were characterized by FTIR using a Nicolet 6700 FTIR spectrometer (Thermo Scientific) in attenuated total reflection (ATR) mode. Spectra were recorded from 4000 to 400 cm^−1^ as the average of 64 scans at room temperature.

#### 2.6.2. Thermogravimetric Analysis (TGA)

The thermal stability of the films and the extract was studied by a TGA-50 SHIMADZU thermogravimetric analyzer (Kyoto, Japan). The analysis was carried out under a nitrogen atmosphere (50 mL/min flow rate), from room temperature to 700 °C at 10 °C/min.

### 2.7. Release Kinetics and Antioxidant Activity of TPS Films

To evaluate polyphenol release, 1 cm^2^ film samples were immersed in 5 mL of 95% ethanol (EU Regulation No. 10/2011) under magnetic stirring in 10 mL amber glass bottles [25,26,27]. At predetermined times, 50 µL aliquots were collected from the supernatant. Experiments were performed in duplicate. The antioxidant activity of each aliquot was determined using ABTS and DPPH assays, as described before, and expressed as percentage inhibition.

TPC was determined using the Folin–Ciocalteu assay following [28]. Analyses were performed in 96-well microplates by mixing 25 µL of sample with 125 µL of the Folin–Ciocalteu reagent (1:10, *v*/*v*). After 3 min, 100 µL of Na_2_CO_3_ were added, and the mixture was incubated for 2 h at room temperature in the dark. Absorbance was measured at 765 nm, and blanks were prepared using distilled water instead of the sample. A calibration curve (5–150 µg/mL) was constructed using gallic acid standards prepared by serial dilution. TPC was expressed as gallic acid equivalents (µg GA/mL).

Release kinetics were evaluated by calculating M(t)/M_inf_, where M(t) is the TPC at time t and M_inf_ the value at equilibrium. The release process was modeled using Fick’s second law in one dimension (Equation (4)) (assuming a semi-infinite system), which is one of the most widely used approaches to describe the migration of active compounds from polymeric matrices into external media [27,29]. The diffusion coefficient (D) was estimated using the minimized scalar routine implemented in SciPy (version 1.12.0). The equation provides the mathematical solution of the law for a semi-infinite system, where L_p_ is the film thickness.

(4) $$\frac{M(t)}{M_{\inf}}=1-\sum_{n=0}^{\infty }\frac{8}{{\left(2n+1\right)}^{2}\pi^{2}}e^{-\frac{{\left(2n+1\right)}^{2}\pi^{2}\mathrm{Dt}}{L_{p}^{2}}}$$

### 2.8. Statistical Analysis

RSM analysis, multi-response optimization using the desirability function, and figure generation were performed with Design-Expert 11 (Stat-Ease, Minneapolis, MN, USA). Model coefficients were estimated by backward multiple regression. Statistical significance was assessed by ANOVA (*p* < 0.05), with a non-significant lack of fit required (*p* > 0.05). Only significant variables (*p* < 0.05) were retained, and model performance was evaluated using the adjusted coefficient of determination (R^2^_adj_). Antioxidant activity data were fitted to the Weibull model using GraphPad Prism 8.

## 3. Results and Discussion

### 3.1. Phenolic Profiles of BSG Extracts

Table 1 shows the compounds tentatively identified by LC-MS/MS in BSG extracts obtained by PLE. Thirteen phenolic compounds were detected, with phenolic acids as the predominant class. Among them, three were hydroxybenzoic acids and five were hydroxycinnamic acids. Gallic acid (C1) was confirmed using a commercial standard. Protocatechuic acid (C2) was assigned based on its *m*/*z* 154 and a fragment at *m*/*z* 109 [30]. Hydroxybenzoic acid (C3, *m*/*z* 137) showed a typical fragment at *m*/*z* 93 resulting from CO_2_ loss [31].

Regarding hydroxycinnamic acids, caffeic acid (C4) and ferulic acid (C9) were confirmed with standards. A compound with *m*/*z* 341 was identified as a caffeic acid hexoside (C5) due to the fragment at *m*/*z* 179 [32,33] and it was the most abundant compound detected. Caffeoylquinic acid (C6, *m*/*z* 353) was assigned based on fragments at *m*/*z* 191 and 179 [34], while p-coumaric acid (C8) was identified according to its characteristic fragmentation pattern [35].

In addition, three flavonols and one flavone were identified. Catechin (C7), quercetin (C10), and apigenin (C11) were confirmed using commercial standards. Finally, compounds with *m*/*z* 354 and 361 were tentatively assigned as xanthohumol (C12) and humulone (C13), respectively, based on their characteristic fragments [21,36].

After identification, the compounds were quantified using the LC-MS/MS database. Seven of the thirteen compounds were quantifiable across the 28 PLE experiments (Table S1). Phenolic acids were the dominant group, with concentrations ranging from 1 to 5 mg/g dw, whereas flavonoids were present at lower levels (0.002–0.05 mg/g dw). Caffeic acid (C4) and its hexoside (C5) were the major compounds, accounting for 86.2–99.9% of the total detected phenolics, depending on the extraction conditions. These findings are in line with previous studies. For instance, Petrón et al. [37] reported 1.979 mg/g dw of caffeic acid in aqueous BSG extracts, a value within the range observed in the present work.

### 3.2. Optimized PLE Conditions

Table 2 shows the mean values of the response variables, extraction yield (Y_1_) and total phenolic content (Y_2_), obtained under the 28 experimental conditions defined by the RSM design matrix. The experimental data were fitted to a polynomial model (Equation (1)), and the corresponding regression coefficients are shown in Table 3. Both models were highly significant (*p* < 0.0001), with adjusted coefficients of determination (R^2^_adj_) between 0.72 and 0.78. No significant lack of fit was detected (*p* > 0.05), indicating that the models adequately describe the experimental data. These results confirm that the models had moderate predictive accuracy.

#### 3.2.1. Effect of PLE Conditions on Response Variables

**Figure 1:**
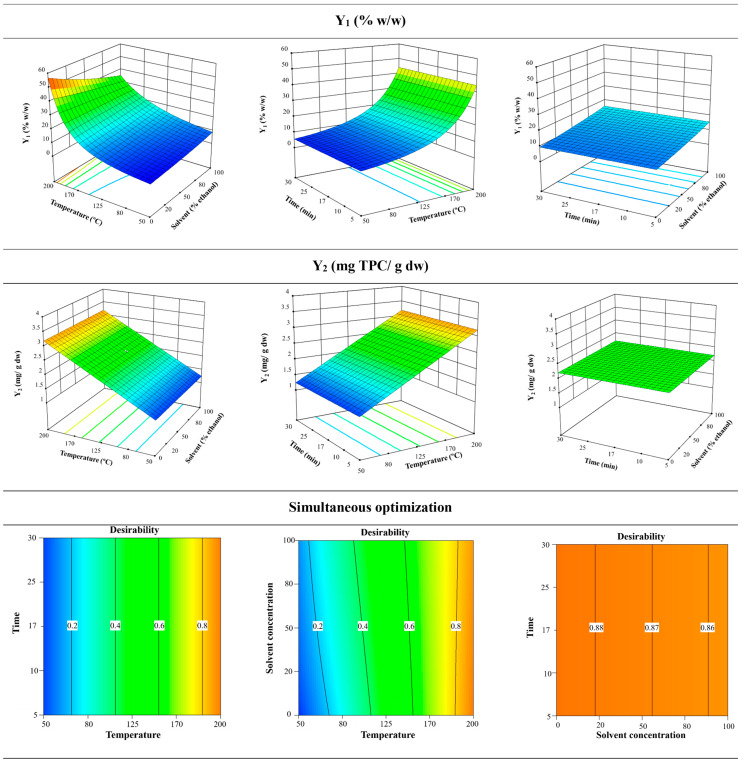
Response surface plots and desirability function for the optimization of BSG phenolic extraction using PLE.

Figure 1 presents the response surface plots obtained from the RSM analysis of BSG. According to the statistical model (Table 3), temperature and solvent composition were the main factors affecting extraction yield (Y_1_). Because an inverse square root transformation was applied, negative coefficients indicate positive effects on the original response.

Accordingly, the negative coefficient for temperature reflects a marked positive effect on extraction yield, with higher temperatures leading to increased yields (Figure 1). Solvent composition also showed a positive effect, indicating that a higher ethanol content slightly enhanced extraction. However, the interaction between temperature and solvent composition was significant (*p* < 0.01) and negatively affected the response. This suggests that the simultaneous use of high temperature and high ethanol concentration reduced yield compared with the effect of each factor individually. In contrast, extraction time was not significant (*p* > 0.05).

For TPC (Y_2_), only the linear effect of temperature was significant and positive, indicating higher phenolic contents at increased temperatures (Table 3, Figure 1). This result suggests that the phenolic compounds extracted are relatively stable within the temperature range studied. Similar trends have been reported for ultrasound-assisted extraction of BSG [10].

The positive influence of temperature can be attributed to enhanced solubility and diffusion, together with reduced solvent viscosity and surface tension. Moreover, higher temperatures weaken interactions between phenolics and the matrix, facilitating their release [9]. However, excessively high temperatures may promote compound degradation [38]. In agreement with previous studies, extraction time had little effect on either yield or TPC [9,39].

#### 3.2.2. Optimal Conditions and Validation

The desirability function analysis defined the optimal conditions for simultaneously maximizing extraction yield and total phenolic content (TPC). These conditions corresponded to an extraction time of 5 min, a temperature of 200 °C, and an ethanol concentration of 0%, meaning water was used as the only solvent (Figure 1). Under these conditions, the model predicted a yield of 42.05% and a TPC of 3.19 mg/g dw, based on Equation (1). Although 200 °C was optimal within the studied experimental range and for the phenolic compounds quantified by LC-MS/MS, the possible degradation or transformation of non-target or highly thermolabile phenolics cannot be completely ruled out.

To validate the model predictions, experiments were carried out independently. The experimental results showed a yield of 37.85 ± 1.37% (*w*/*w*) and a TPC of 2.54 ± 0.70 mg/g dw. Statistical analysis revealed no significant differences between predicted and experimental values for extraction yield (*p* > 0.05). In contrast, the experimentally obtained TPC was slightly lower than the predicted value, although it remained within the same order of magnitude. This difference may be partly explained by the moderate predictive ability of the model (R^2^ = 0.72), together with matrix heterogeneity and potential LC-MS/MS matrix effects. Importantly, the TPC values obtained are comparable to those reported using other extraction techniques [10,37], which supports the validity of the results. These findings confirm the moderate reliability of the developed models for predicting TPC recovery from BSG.

### 3.3. Antioxidant Activity of Phenolic-Rich Extract from BSG

**Figure 2:**
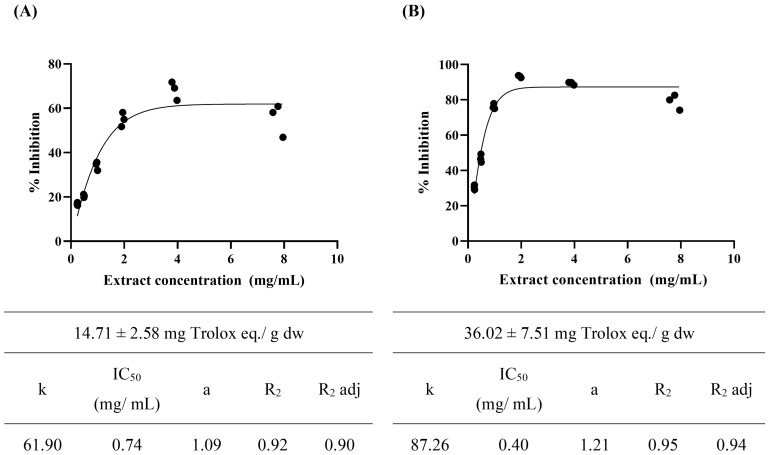
Weibull model fitting of antioxidant activity curves, including statistical parameters obtained from nonlinear regression for (**A**) DPPH and (**B**) ABTS radicals. Antioxidant activity expressed as Trolox equivalents (mg/g dw) for both methods is also included.

Figure 2 shows the antioxidant activity of the BSG extract obtained under the optimal PLE conditions, evaluated using the DPPH and ABTS assays. The experimental data were fitted to a nonlinear Weibull model, showing a good fit, with R^2^_adj_ of 0.90 for DPPH and 0.94 for ABTS.

From these models, IC_50_ values of 0.74 mg/mL (DPPH) and 0.40 mg/mL (ABTS) were obtained, indicating a stronger scavenging effect against the ABTS radical. This difference is expected, since the ABTS assay can detect both hydrophilic and lipophilic antioxidants, while DPPH mainly reacts with hydrogen-donating compounds in less polar conditions. As expected, Trolox, used as a reference, showed much lower IC_50_ values (0.055 mg/mL for DPPH and 0.046 mg/mL for ABTS). This is consistent with its nature as a pure, low-molecular-weight antioxidant. In comparison, the BSG extract showed IC_50_ values about one order of magnitude higher, which is reasonable given that it is a complex mixture of phenolic acids and other bioactive compounds, rather than a single compound. Even so, the extract showed a clear antioxidant capacity, especially in the ABTS assay, suggesting that it can act against different types of radicals. This is in line with the phenolic profile of BSG, which includes compounds that can act through different mechanisms, such as hydrogen atom transfer and electron donation.

To allow comparison with other studies, the antioxidant activity was also expressed as TEAC. The values obtained here were notably higher than those reported for BSG extracts using other extraction methods. For example, Naibaho et al. [40] reported ABTS values between 0.225 and 0.75 mg Trolox/g dw using autoclave extraction. Similarly, Iadecola et al. [10] found 0.42 mg Trolox eq/g dw (DPPH) and 5.82 mg Trolox eq/g dw (ABTS) with ultrasound-assisted extraction. In contrast, the extract obtained in this study reached 36.02 mg Trolox eq/g dw in the ABTS assay, showing a much higher recovery of antioxidant compounds with PLE.

This higher activity can be explained by the PLE conditions. The combination of high temperature and pressure (around 1600 psi) improves solvent penetration, increases the solubility of phenolic compounds, and enhances mass transfer. As a result, more bound compounds can be released from the lignocellulosic matrix. In addition, the use of RSM helped to identify the best extraction conditions, further improving the recovery. These results suggest that combining PLE with process optimization is an effective way to valorize BSG as a source of antioxidant compounds.

### 3.4. Structural Analysis of TPS Film Containing Extract

**Figure 3:**
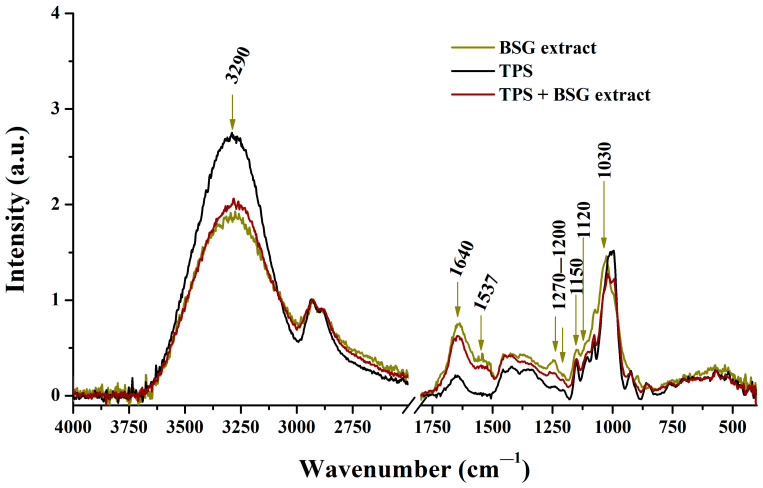
FTIR analysis of BSG extract, TPS film and TPS film containing extract.

The FTIR spectrum of the phenolic extract obtained from BSG (Figure 3) showed the typical absorption bands associated with phenolic compounds. A band around 1640 cm^−1^ can be linked to C=O stretching of carboxyl groups. In addition, bands in the 1606–1588 cm^−1^ range and at 1537 cm^−1^ correspond to C=C stretching in the aromatic ring. These features are commonly reported for hydroxybenzoic acids and related phenolic compounds and are consistent with previous studies [41,42,43]. A broad band centered at about 3290 cm^−1^ was also observed, which is typical of O-H stretching from phenolic and carboxylic hydroxyl groups. The presence of two close peaks between 1200 and 1270 cm^−1^ can be associated with O-H bending of hydroxyl groups attached to the aromatic ring, something that has been linked to caffeic acid derivatives [44]. Bands at 1150 and 1030 cm^−1^ were assigned to C-O stretching of alcohol groups, while signals below 1120 cm^−1^ are related to C-C bending in the aromatic ring [45].

The FTIR spectrum of the neat TPS film showed the expected features of a starch-based material. A broad band between 3200 and 3400 cm^−1^ was observed, corresponding to O-H stretching from hydroxyl groups in starch and from bound water in the matrix. Signals in the 2800–3000 cm^−1^ region were linked to C-H stretching of the polysaccharide chains. In addition, bands at 920, 1020, and 1152 cm^−1^ were assigned to C-O stretching in the glucose rings, which are characteristic of starch [46,47].

After adding the BSG phenolic extract to the TPS matrix, the FTIR spectrum of the resulting film showed a combination of both components. The main starch bands were still clearly visible, suggesting that the polymer structure was not altered. At the same time, some changes in intensity and slight shifts were observed, especially in the 1500–1700 cm^−1^ region. This area is related to carbonyl and aromatic vibrations, which indicates the presence of phenolic compounds in the matrix.

A slight decrease and broadening of the O–H band around 3290–3400 cm^−1^ was also observed compared to neat TPS. This suggests that hydrogen bonding interactions may be taking place between the hydroxyl groups of starch and those of the phenolic compounds.

No new absorption bands appeared in the TPS + extract spectrum, which would indicate the formation of new covalent bonds. This suggests that the interaction between the extract and the TPS matrix is mainly physical, driven by hydrogen bonding and dipole–dipole interactions. These results confirm that the phenolic extract was successfully incorporated into the TPS matrix, with good compatibility and without damaging the structure of either component. These results suggest that TPS-based films incorporating BSG extracts may hold promising potential as functional materials.

### 3.5. Thermal Stability of TPS Film Containing BSG Phenolic Extract

**Figure 4:**
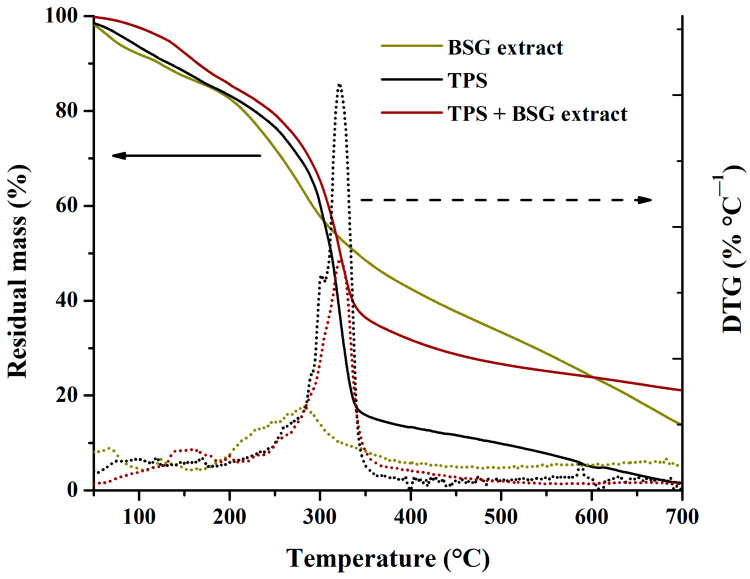
Residual mass and DTG curves of BSG extract, TPS film and TPS film containing extract. Solid lines correspond to residual mass (%) and are read on the left y-axis; dotted lines correspond to DTG (% °C^−1^) and are read on the right y-axis.

Figure 4 shows the TGA and DTG curves of the BSG extract, the neat TPS film, and the TPS film with the phenolic extract. The BSG extract showed low thermal stability, as it started to degrade at lower temperatures than the TPS films. The DTG curve suggests that this degradation occurs in several steps, which can be attributed to the fact that the extract is a mixture of compounds with different sizes and stabilities. Mass loss occurred gradually up to 700 °C, with the main degradation phase between 150 and 400 °C. This range is related to the degradation of low-molecular-weight phenolics, organic acids, and related compounds such as hydroxybenzoic and hydroxycinnamic acid derivatives [48,49]. At higher temperatures, the mass loss is more gradual, suggesting slow decomposition and partial carbonization of aromatic structures.

The TPS film without extract had the typical three-stage degradation pattern reported for plasticized starch materials. The first stage, observed between 50 and 200 °C, is mainly due to the loss of water and glycerol. The second stage, with a maximum around 322 °C, corresponds to the breakdown of the starch matrix. This involves processes such as depolymerization, dehydration, and the cleavage of glycosidic bonds, with some condensation reactions [50,51,52,53]. The third stage, above 500 °C, is linked to the degradation of the remaining structure and the formation of carbonaceous residues. Some aromatic structures are formed at 600 °C, and eventually turn into amorphous carbon, leaving a relatively low residual mass, which is typical for starch-based materials [54].

In TPS film containing the BSG extract, the overall degradation profile remained similar to that of the TPS film alone, especially during the main degradation stage. The maximum degradation temperature did not change significantly, which suggests that adding the extract does not negatively affect the main thermal stability of the material. This is important from a processing point of view, since it means the film can still resist typical thermal conditions. However, some differences can be observed at higher temperatures. The TPS film with the extract showed a noticeably higher residual mass compared to neat TPS, indicating greater char formation. This can be explained by the presence of phenolic compounds with aromatic structures, which tend to form more stable carbon residues during heating. In addition, the hydrogen bonding interactions between the phenolic compounds and the starch chains, as suggested by the FTIR results, may form a more compact and thermally stable structure.

From these results, adding the BSG phenolic extract could not change the main degradation behavior of TPS, but it would improve its performance at higher temperatures by increasing the amount of residue formed. This supports the hypothesis that the extract is well incorporated into the matrix and may offer advantages in applications in biodegradable starch-based films with better thermal resistance.

### 3.6. Release Kinetics of TPS Film

The release of phenolic compounds from the TPS film depends on the polymer matrix, the properties of the BSG extract (polarity), and the interactions between the extract and the polymeric matrix and the extract with the medium. These factors influence the diffusion behavior of the bioactive compounds and thus, their performance under real conditions when used as ingredients in active packaging.

**Figure 5:**
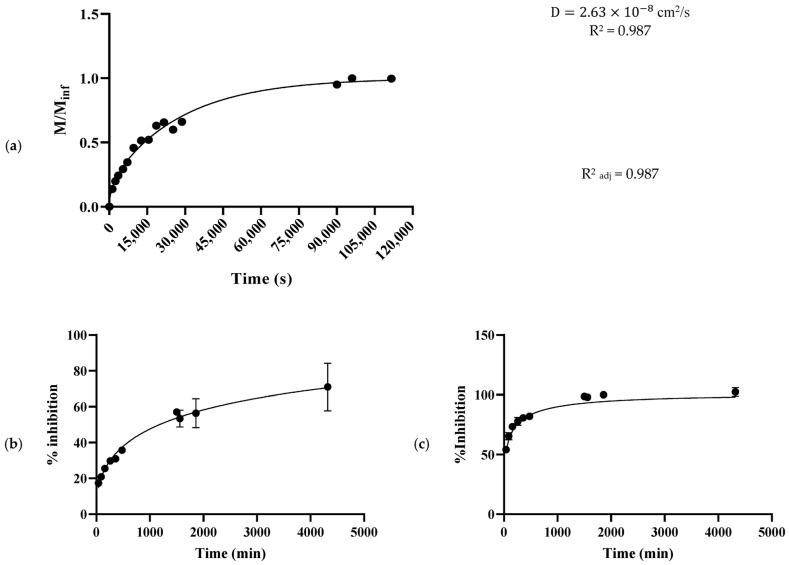
Release kinetics and antioxidant activity of phenolic compounds from TPS films in 95% ethanol. (**a**) Fractional release (M/M_inf_) as a function of time and fitted diffusion model; percentage of inhibition during the release test against (**b**) DPPH and (**c**) ABTS radicals.

Figure 5a shows the fractional release (M/M_inf_) of phenolic compounds from the TPS film in 95% ethanol. The amount of phenolics in the medium, measured using the Folin–Ciocalteu method, increased over time until it reached equilibrium. The release followed a two-step pattern with a fast initial release, referred to as a “burst effect”, followed by a slower diffusion-controlled stage. The equilibrium was reached after 1500 min. The initial fast release can be attributed to phenolic compounds located near the film surface, which are more easily released. In contrast, the slower stage is related to the diffusion of compounds trapped inside the polymer matrix.

The experimental data were well described by Fick’s second law of diffusion (Equation (4)), as shown by the fitted curve in Figure 5a. The calculated diffusion coefficient was D = 2.63 × 10^−8^ cm^2^/s, with a high R^2^ value (0.987), indicating a very good fit. This model makes some assumptions such as simplified system, including one-dimensional diffusion, constant diffusion conditions, and no significant changes in the polymer (such as degradation or swelling) during the experiment. Under these assumptions, the model provided a good description of the release behavior.

The diffusion coefficient was in the same range, although slightly higher, than values reported for other TPS systems with phenolic compounds. For example, Miao et al. [55] found lower values for films containing tea polyphenols, which they linked to the use of porous starch. In that case, the more complex structure slows down diffusion. In contrast, the system used in this work allows for faster release, which could be useful in applications where a quick antioxidant effect is needed. It is important to note that the present study was conducted using 95% ethanol, as recommended by EU regulations for fatty food applications. Nevertheless, it should be noted that these results represent a preliminary assessment and may not fully reflect release behavior under real food conditions, where factors such as moisture content, fat content, pH, and storage environment may significantly influence diffusion. Investigating the release behavior in aqueous and low-alcohol food simulants would be valuable in future studies to provide a more comprehensive assessment of the material’s performance across a broader range of food packaging applications.

To better understand the practical impact of this release, the antioxidant activity of the released compounds was also evaluated using DPPH and ABTS assays (Figure 5b,c). The inhibition increased with time, following a trend similar to the release profile. A strong antioxidant effect was observed at the beginning, in line with the burst release, and then gradually stabilized as equilibrium was reached. The ABTS assay showed higher inhibition values than DPPH, attributed to its higher sensitivity to a wider range of phenolic compounds. Thus, these results suggest that the developed TPS films may serve as proof-of-concept active materials with antioxidant properties. This is particularly relevant for food packaging applications involving oxidation-sensitive products, where such materials could potentially contribute to slowing down oxidative processes.

## 4. Conclusions

This work shows the potential of BSG as a source of antioxidant compounds for the development of bioactive starch-based materials. PLE was optimized using RSM, and the best conditions were identified as 5 min, 200 °C, and water as the sole solvent. To the best of our knowledge, this is the first application of PLE for recovering phenolic compounds from BSG. The use of water as extraction solvent avoids organic solvents, reduces downstream processing requirements, and supports the environmental compatibility of the process. Although 200 °C was optimal within the studied experimental range and for the phenolic compounds quantified by LC-MS/MS, the possible degradation or transformation of non-target or highly thermolabile phenolics cannot be completely ruled out.

The antioxidant-rich extract obtained under the optimized conditions was successfully incorporated into TPS films, providing active functionality to the material. The films showed strong antioxidant activity in the release medium, reaching approximately 70% inhibition in the DPPH assay and complete inhibition in the ABTS assay after 3 days in 95% ethanol. These results indicate that BSG-derived phenolic compounds can be incorporated into TPS matrices and released into a food simulant, supporting their potential use as active ingredients in biodegradable materials.

Overall, this study contributes to the valorization of BSG within a circular economy approach through the green extraction of bioactive compounds and their incorporation into functional starch-based films. However, these materials should be considered proof-of-concept active films rather than fully validated packaging systems. Future work should focus on optimizing extract incorporation, evaluating performance in different food systems, assessing biodegradation, and addressing BSG variability, extract stability, and techno-economic feasibility to support practical industrial implementation.

## Figures and Tables

**Table 1:** Tentative identification of phenolic compounds in BSG extracts by LC-MS/MS.

	Compound	Group	Chemical Formula	RT (min)	M-H (*m*/*z*)	Transition ^1^ (Collision Energy ^2^)
C1	Gallic Acid	Hydroxybenzoic acid	C_7_H_6_O_5_	5	169	125 (15), 79 (24)
C2	Protocatechuic acid	Hydroxybenzoic acid	C_7_H_6_O4	5	154	109 (45), 153 (30)
C3	Hydroxybenzoic acid	Hydroxybenzoic acid	C_7_H_6_O_3_	5.5	137	93 (15), 109 (15)
C4	Caffeic acid	Hydroxycinnamic acid	C_9_H_8_O_4_	5.7	179	161 (6), 58 (16)
C5	Caffeic acid hexoside	Hydroxycinnamic acid	C_15_H_18_O_9_	5.7	341	179 (13), 113 (20)
C6	Caffeoylquinic acid	Hydroxycinnamic acid	C_9_H_8_O_4_	7	353	119 (24), 179 (24)
C7	Catechin	Flavonol	C_15_H_14_O_6_	14.5	289	203 (19), 109 (26)
C8	P-coumaric acid	Hydroxycinnamic acid	C_9_H_8_O_3_	17	163	119 (14), 93 (31)
C9	Ferulic acid	Hydroxycinnamic acid	C_10_H_10_O_4_	20	193	178 (12), 134 (17)
C10	Quercetin	Flavonol	C_15_H_10_O_7_	26	301	178 (18), 151 (22)
C11	Apigenin	Flavone	C_15_H_10_O_5_	28	269	117 (35), 269 (20)
C12	Xanthohumol	Prenylflavonoids	C_21_H_22_O_5_	32	354	119 (25), 353 (15)
C13	Humulone	Prenylated phloroglucinol derivative	C_21_H_30_O_5_	40	361	292 (20), 362 (10)

^1^ *m*/*z*, ^2^ Voltage.

**Table 2:** Experimental design matrix showing the actual levels of the independent variables and the resulting response variables Y_1_ (yield, g extract/100 g dw) and Y_2_ (total polyphenolic content in mg TP/g dw).

Run	Factors			Response Variables	
	t (min)	T (°C)	S (% Ethanol in Water)	Y_1_ (% *w*/*w*)	Y_2_ (mg TP/g dw)
1	10.1	80.4	20.3	5.32	1.06
2	10.1	80.4	79.7	7.75	1.35
3	10.1	169.6	20.3	18.34	4.97
4	10.1	169.6	79.7	47.66	3.01
5	24.9	80.4	20.3	5.41	1.92
6	24.9	80.4	79.7	10.67	2.29
7	24.9	169.6	20.3	27.92	3.24
8	24.9	169.6	79.7	54.373	2.82
9	30	125	50	42.45	1.93
10	5	125	50	9.09	1.35
11	17.5	50	50	5.414	1.46
12	17.5	200	50	47.47	1.92
13	17.5	125	0	7.73	1.89
14	17.5	125	100	12.04	1.66
15	5	50	0	3.74	1.45
16	5	50	100	7.12	1.43
17	5	200	0	32.98	3.58
18	5	200	100	10.82	2.93
19	30	50	0	3.61	1.36
20	30	50	100	7.45	1.09
21	30	200	0	25.28	3.33
22	30	200	100	23.85	3.15
23	17.5	125	50	12.77	2.11
24	17.5	125	50	11.25	2.62
25	17.5	125	50	11.89	2.02
26	175	125	50	11.40	1.82
27	17.5	125	50	12.54	3.10
28	17.5	125	50	7.82	2.21

For response Y_2_ (TPC), Runs 3 and 12 were excluded from the RSM fitting after diagnostic evaluation of the initial model including all experimental points. The exclusion was based on residual and influence diagnostics, including externally studentized residuals, residuals-versus-predicted plots, normal probability plots of residuals, Box–Cox analysis and Cook’s distance.

**Table 3:** Statistical parameters and the regression coefficients obtained for each model in terms of coded factors.

RSM Models	Y_1_	Y_2_
*Statistical parameters*		
Model significance (*p*-value)	<0.0001	<0.0001
Lack of fit (*p*-value)	0.08	0.75
R^2^	0.80	0.73
R^2^_adj_	0.78	0.72
Transformation	Inverse Sqrt	None
*Regression coefficients*		
Intercept (β_0_)	0.30 ± 0.01	2.22 ± 0.08
Lineal		
β_1_ (t)	NS	NS
β_2_ (T)	−0.13 ± 0.14 ^a^	0.97 ± 0.12 ^a^
β_3_ (S)	−0.03 ± 0.14 ^c^	NS
Interactive		
β_12_	NS	NS
β_13_	NS	NS
β_23_	0.05 ± 0.01 ^b^	NS

Y_1_: yield in g extract/100 g dw; Y_2_: total polyphenolic content in mg TP/g dw; a: *p* < 0.0001, b: *p* < 0.01, c: *p* < 0.05, NS: non-significant (*p* > 0.05).

## Data Availability

The original contributions presented in this study are included in the article/Supplementary Materials. Further inquiries can be directed to the corresponding author.

## Supplementary Materials

The following supporting information can be downloaded at: https://www.mdpi.com/article/10.3390/foods15122141/s1, Table S1: Phenolic compounds (mg/g dw) quantified in the 28 experiments included in the experimental design matrix for the PLE extraction of BSG.
