# Supplementary material for: Valorization of Brewing By-Products for Sustainable Active Material
Source: Foods. 2026 Jun 13;15(12):2141. doi: 10.3390/foods15122141 (PMC13298428; doi:10.3390/foods15122141)
Supplement: Supplementary file 1 [file foods-15-02141-s001.zip › foods-4337355-supplementary.pdf]

Table S1. Phenolic compounds (mg/g dw) quantified in the 28 experiments included in the experimental design matrix for the PLE extraction of BSG.

	**Phenolic compounds (mg / g dw)**						
Run	**C1**	**C4**	**C5**	**C7**	**C9**	**C10**	**C11**
**1**	0.0002	0.1325	0.9272	0.0013	0.0000	0.0000	0.0003
**2**	0.0042	0.2192	1.1085	0.0044	0.0085	0.0002	0.0010
**3**	0.0008	0.8193	3.9222	0.0112	0.2167	0.0013	0.0017
**4**	0.0021	0.5128	2.4923	0.0035	0.0000	0.0000	0.0021
**5**	0.0017	0.3084	1.6107	0.0012	0.0000	0.0000	0.0009
**6**	0.0018	0.3647	1.9235	0.0013	0.0000	0.0000	0.0009
**7**	0.0025	0.5346	2.6957	0.0045	0.0000	0.0000	0.0023
**8**	0.0098	1.6191	1.1882	0.0035	0.0000	0.0000	0.0012
**9**	0.0054	0.3653	1.3449	0.0103	0.1967	0.0012	0.0010
**10**	0.0021	0.1739	1.1279	0.0060	0.0349	0.0006	0.0003
**11**	0.0012	0.3558	1.0752	0.0091	0.0146	0.0000	0.0004
**12**	0.0002	0.2451	1.6686	0.0030	0.0000	0.0005	0.0005
**13**	0.0128	0.4614	1.3523	0.0215	0.0437	0.0000	0.0008
**14**	0.0006	0.2566	1.3441	0.0247	0.0329	0.0010	0.0004
**15**	0.0011	0.2512	1.1898	0.0097	0.0000	0.0000	0.0006
**16**	0.0016	0.4609	0.7676	0.0033	0.1925	0.0000	0.0005
**17**	0.0031	0.6366	2.8636	0.0239	0.0472	0.0012	0.0005
**18**	0.0016	0.4928	2.4263	0.0062	0.0000	0.0000	0.0003
**19**	0.0013	0.2363	1.1189	0.0075	0.0000	0.0000	0.0002
**20**	0.0008	0.1655	0.8952	0.0194	0.0103	0.0007	0.0003
**21**	0.0037	0.5522	2.7528	0.0197	0.0000	0.0000	0.0012
**22**	0.0016	0.7921	2.0078	0.0540	0.2955	0.0000	0.0011
**23**	0.0006	0.2799	1.8131	0.0139	0.0000	0.0005	0.0004
**24**	0.0023	0.4609	2.1459	0.0092	0.0000	0.0000	0.0008
**25**	0.0002	0.3083	1.7041	0.0048	0.0000	0.0004	0.0005
**26**	0.0008	0.2322	1.5373	0.0223	0.0212	0.0004	0.0003
**27**	0.0013	0.6423	2.4214	0.0294	0.0000	0.0020	0.0011
**28**	0.0006	0.3834	1.8067	0.0168	0.0000	0.0015	0.0008
C1: Gallic Acid; C2: Protocatechuic acid; C3: Hydroxybenzoic acid; C4: Caffeic acid; C5: Caffeic acid hexoside; C6: Caffeoylquinic acid; C7: Catechin; C8: P-coumaric acid; C9: Ferulic acid; C10: Quercetin; C11: Apigenin; C12: Xanthohumol; C13: Humulone							
